# Identification of the principal transcriptional regulators for low-fat and high-fat meal responsive genes in small intestine

**DOI:** 10.1186/s12986-017-0221-3

**Published:** 2017-10-23

**Authors:** Octave Mucunguzi, Aicha Melouane, Abdelaziz Ghanemi, Mayumi Yoshioka, André Boivin, Ezequiel-Luis Calvo, Jonny St-Amand

**Affiliations:** 1 CREMI, CHU de Québec Research Center, Quebec, QC G1V 4G2 Canada; 2 Department of Molecular Medicine, Faculty of Medicine, Laval University, Quebec, QC G1V 0A6 Canada; 3 Functional Genomics Laboratory, CREMI, CHUL-CHU de Québec Research Center, 2705 Boul. Laurier, Québec, PQ G1V 4G2 Canada

**Keywords:** Low-fat diet, High-fat diet, Duodenum, Mucosa, Serial analysis of gene expression, Microarray

## Abstract

**Background:**

High-fat (HF) diet is a well-known cause of obesity. To identify principle transcriptional regulators that could be therapeutic targets of obesity, we investigated transcriptomic modulation in the duodenal mucosa following low-fat (LF) and HF meal ingestion.

**Methods:**

Whereas one group of mice was sacrificed after fasting, the others were fed ad libitum with LF or HF meal, and sacrificed 30 min, 1 h and 3 h after the beginning of the meal. A transcriptome analysis of the duodenal mucosa of the 7 groups was conducted using both microarray and serial analysis of gene expression (SAGE) method followed by an Ingenuity Pathways Analysis (IPA).

**Results:**

SAGE and microarray showed that the modulation of a total of 896 transcripts in the duodenal mucosa after LF and/or HF meal, compared to the fasting condition. The IPA identified lipid metabolism, molecular transport, and small molecule biochemistry as top three molecular and cellular functions for the HF-responsive, HF-specific, HF-delay, and LF-HF different genes. Moreover, the top transcriptional regulator for the HF-responsive and HF-specific genes was peroxisome proliferator-activated receptor alpha (PPARα). On the other hand, the LF-responsive and LF-specific genes were related to carbohydrate metabolism, cellular function and maintenance, and cell death/cellular growth and proliferation, and the top transcriptional regulators were forkhead box protein O1 (FOXO1) and cAMP response element binding protein 1 (CREB1), respectively.

**Conclusions:**

These results will help to understand the molecular mechanisms of intestinal response after LF and HF ingestions, and contribute to identify therapeutic targets for obesity and obesity-related diseases.

**Electronic supplementary material:**

The online version of this article (10.1186/s12986-017-0221-3) contains supplementary material, which is available to authorized users.

## Background

High-fat (HF) diet contributes to increase daily energy intake and body fatness [1, 2]. Thus, controlling fat intake is an important determinant within the etiology of obesity. It has been reported that some protective mechanisms against diet-induced obesity are blunted after an establishment of obesity [3, 4]. Therefore, the study of established obesity may not reveal the primary cause which has led to its development which makes it important to acquire knowledge on the initial events responsible for the development of obesity. In order to identify peripheral signals (appetite and satiety signals from the digestive tract to the central nervous system) that can be therapeutic targets of obesity, we have already investigated transcriptomic changes in the duodenum mucosa after a HF or low-fat (LF) meal ingestion using the serial analysis of gene expression (SAGE) method [5].

Gene profiling approaches allow gaining global insights into the transcriptome. Commercial software is now widely available to analyze relevant functions, pathways, networks and transcriptional regulators. Several studies attempted to characterize the intestinal transcriptional responses after a HF diet by using microarrays [6–11], but all used a LF diet as a control. Therefore, no transcriptional information is available regarding the intestinal responses to the LF diet. Moreover, results obtained by SAGE and microarray in previous studies were only partially comparable, and some authors have suggested that these approaches may be rather complementary to each other in the study of transcriptome [12, 13].

In order to identify the principal transcriptional regulators for LF- and HF-meal responsive genes, which could be therapeutic targets for obesity and related diseases, the present study has used our previous data with SAGE method [5], as well as data obtained by microarray. Then, we have analyzed the relevant pathways, networks and transcriptional regulators using the Ingenuity Pathways Analysis (IPA).

## Methods

### Animals, diet and samples preparation

Detailed experimental procedures including amount of energy and macronutrients ingested have been reported elsewhere [5]. Briefly, a total of 140 male C57BL6 mice (12 wks-old, 24.5 ± 2.2 (mean ± SD) g body weight, Charles River Canada Inc., St Constant, QC, Canada) were fed a LF diet (Research Diet # 12450B: 10% calories from fat, New Brunswick, NJ, USA) for two wks, fasted for 12 h, and randomly distributed into seven groups (20 mice per group). One group of fasted mice was sacrificed (fasting group) in the morning, whereas the remaining six groups were fed ad libitum with a LF or HF (Research Diet # 12492: 60% calories from fat) meal until they were sacrificed 30 min, 1 h and 3 h after the beginning of the meal (Six groups: LF30min, LF1h and LF3h, HF30min, HF1h, and HF3h). Each five mice per group were assigned for the sacrifice per day (9 h 00 - 12 h 00), and a mouse from each group was randomly sacrificed during each 35 min. The fasting/meal starting time of each mouse was adjusted according to the assigned group. Immediately after the sacrifice, duodenum (first 5 cm of small intestine) was opened vertically, flushed clean with saline, and the mucosa was removed by scrapping with a glass microscope slide. The samples were rapidly collected and snap frozen in liquid nitrogen and stored at -80 °C until total RNA and protein extractions.

### Total RNA preparation

Total RNA, isolated from pooled duodenum mucosa for each group (*n* = 20) by Trizol (Life Technologies Inc., Burlington, ON, Canada) [5], was used for both SAGE and microarray analysis. The quality of total RNA was monitored by micro-capillary electrophoresis (Bioanalizer 2100, Agilent Technologies, Mississauga, ON, Canada).

### SAGE analysis

Previously published SAGE data (two-fold change, *P* ≤ 0.05) [5] were used for functional classification, gene expression pattern identification as well as the IPA.

### Microarray analysis

Experiments were performed in duplicate by using pooled RNA from each group of mice. Total RNA (10 μg) was used for cDNA synthesis according to the Affymetrix (Santa Clara, CA, USA) manual. Hybridization to GeneChips MOE 430 v2.0 arrays representing 45,101 transcripts and expressed sequence tags followed by probing and scanning was performed according to the Affymetrix manual. The background subtraction and normalization of probe set intensities were performed using the method of Robust Multiarray Analysis described by Irizarry et al. [14]. To identify differentially expressed genes, gene expression intensity in HF and LF groups was compared to the fasting condition using a moderated *t*-test and a Bayes smoothing approach [15]. To correct the effect of multiple testing, the false discovery rate, was estimated from *p* values derived from the moderated *t*-test statistics [16]. The analysis was performed using the affylmGUI Graphical User Interface for the limma microarray package [17]. Genes were considered to be significantly differentially expressed if *P*-values were <0.05. Under these conditions, a minimal mean ratio of 2 fold was used as threshold for induced or repressed genes by the LF or HF ingestion.

### Validation of gene expressions by quantitative real-time PCR (Q_RT-PCR)

The confirmation of several SAGE data by Q_RT-PCR has already been published [5]. The validation of microarray data is shown in the Additional file 1: Figure S1.

### Functional classification of LF- and/or HF- meal responsive genes

For both the SAGE and microarray data, functional classification of the genes modulated after the LF- and HF ingestions was based upon the genome directory [18] and the OMIM (http://www.ncbi.nlm.nih.gov/) as well as previously published literatures.

Compared to the fasting condition, SAGE detected 369 transcripts and microarray detected 685 other transcripts, all significantly modulated after LF- and/or HF-meal ingestion. These 1054 transcripts classified under 13 functions: Digestion, hormone/peptide, receptor, transport, signaling, RNA/DNA processing, protein metabolism, growth, cytoskeleton, metabolism, homeostasis, immunity, and others/unknown (Additional file 1: Figure S2). The Chi^2^ test was used to identify the significant differences (*P* < 0.05) in the distributions on the total number of transcript species classified under each function.

### Pattern identification of LF- and/or HF-meal responsive genes

**Fig. 1:**
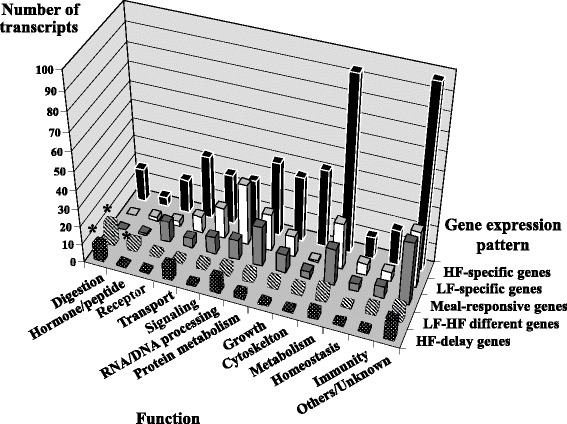
Comparison of the transcripts number detected in each functional classification among five expression patterns. *Significant difference from the expected distribution of number of modulated transcripts (*P* < 0.05). Abbreviations: HF, high-fat; LF, low-fat

After excluding the unknown 158 SAGE transcripts (from the 1054), the remaining 896 transcripts, obtained from both the SAGE and microarray, were classified into six patterns: LF-specific (modulated only in the LF condition), HF-specific (modulated only in the HF condition), meal-responsive (modulated by both HF and LF meals at the same time point and in the same direction), LF-delay (modulated by both HF and LF meals in the same direction but showing delay of modulation in the LF condition), HF-delay (modulated by both HF and LF meals in the same direction but showing delay of modulation in the HF condition), and LF-HF different (modulated by both HF and LF meals but at different time points and/or in opposite direction) patterns. The transcripts in each gene expression pattern were classified into 13 functions except for the LF-delay pattern which contains only 5 genes (Fig. 1). The Chi^2^ test was used to identify the significant differences (*P* < 0.05) in distributions on total number of transcript species classified in each function.

### IPA

Significantly modulated canonical pathways, molecular and cellular functions, networks and transcriptional regulators after the LF and HF meal ingestions were analyzed using the web-based bioinformatics tool, IPA (Ingenuity® Systems, http://www.ingenuity.com). First, data obtained from the SAGE and microarray were separately analysed using the GenBank accession number information in order to compare the difference of detected transcripts between them. Then, these data were combined for the analysis of the LF- and HF- responsive genes using the GenBank accession number information (*n* = 896). The transcripts which were not mapped to the GenBank accession number were excluded (*n* = 129). Only 767 mapped transcripts were then matched in the IPA database. As a result, a total of 629 matched genes were used in our analysis: genes detected by SAGE, genes detected by microarray, LF-responsive genes, HF-responsive genes, LF-specific genes, HF-specific genes, meal-responsive genes, LF-delay genes, HF-delay genes, and LF-HF different genes. For the LF-delay genes analysis, since only four genes were available, their IPA results were excluded.

Fisher’s exact test was used to calculate a *P*-value determining the probability that the association between the genes in the dataset and the function (canonical pathway, molecular and cellular function, network and transcriptional regulator) is explained by chance alone (*P* < 0.001).

### Validation of transcriptional regulator expressions by western blot

The principal transcriptional regulators identified by the IPA were validated by western blot (*n* = 9, 8 and 7 for the fasting, LF-3 h and HF-3 h conditions, respectively). Total proteins were extracted using a RIPA buffer and protease inhibitors cocktail (Sigma-Aldrich Canada Co., Oakville, ON, Canada). Five to 30 μg of proteins were separated by SDS-PAGE using the TGX Stain-Free FastCast acrylamide solutions (Bio-Rad Laboratories Ltd., Mississauga, ON, Canada), and trihalo compound in the gels was activated under UV. Then, total proteins were transferred onto PVDF membrane (Bio-Rad Laboratories Ltd.), and visualized under UV using the AlphaImager™ 1220 (Alpha Innotech Co., San Leandro, CA, USA). Membranes were blocked using the Pierce™ Protein-Free (TBS) blocking buffer (Life Technologies Inc.), incubated with primary (Additional file 2: Table S1) and secondary antibodies (sc-2004 or sc-2005, 1/10000 dilution: Santa Cruz Biotechnology Inc., Dallas, Texas, USA), and visualized with the Clarity™ Western ECL Blotting Substrate on a film (Bio-Rad Laboratories Ltd.). The visualized total proteins on the membranes (loading control) and target proteins on the films were quantified using the ImageJ software. Prior to the western blot, pooled samples were used to determine the quantity of loading proteins (0 - 40 μg) and dilution of primary antibody (1/200-1/9000). The same pooled sample was loaded in each gel, and used as a positive control (PC) to normalize the differences between each membrane. The density of each lane on the membrane (DM) and on the film (DF) was expressed as a ratio to each PC on the same membrane/film. Then, the quantity of protein loaded was normalized by dividing DF by DM, as previously suggested [19]. Results are expressed as mean ± SEM. Differences between diet conditions were evaluated through one-way ANOVA followed by the Fisher’s Protected LSD post hoc tests (*P* < 0.05). In a case of a transcriptional regulator for the meal-responsive genes, *t*-test was used to identify the significant difference (*P* < 0.05) between the fasting condition and fed condition (LF-3 h plus HF-3 h).

## Results

### LF- and HF-responsive genes

**Fig. 2:**
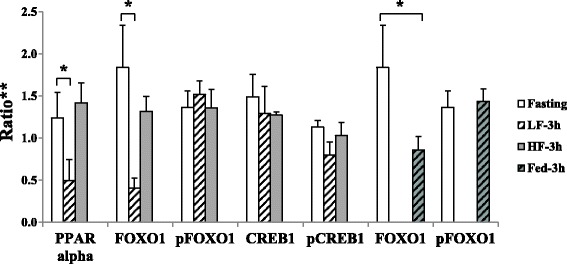
Western blot analysis of transcriptional regulators expressions. *Significant difference between two conditions (*P* < 0.05). **Data are shown as a ratio to the loading control on the same membrane/film and mean ± SEM. Abbreviations: CREB1, cAMP response element binding protein; FOXO1, forkhead box protein O1; HF, high-fat; LF, low-fat; pCREB1, phosphorylated CREB1; pFOXO1, phosphorylated FOXO1; PPAR, peroxisome proliferator-activated receptor

The IPA revealed that the main molecular and cellular functions controlled by the LF-responsive genes were cell death, cellular function and maintenance, and carbohydrate metabolism (Table 1). The top transcriptional regulator was forkhead box protein O1 (FOXO1) (Table 1), and the reduced protein expression after a LF meal was confirmed by western blot (Fig.2). On the other hand, the HF-responsive genes ware related to lipid metabolism, molecular transport, and small molecule biochemistry, and peroxisome proliferator-activated receptor alpha (PPARα) was found to be the top transcriptional regulator (Table 1).

**Table 1:** Comparison of significantly modulated molecular and cellular functions, and transcription regulators after low-fat (LF) and high-fat (HF) meal ingestion

	LF-responsive genes	HF-responsive genes
Top 3 molecular and cellular functions		
1	Cell death	Lipid metabolism
2	Cellular function and maintenance	Molecular transport
3	Carbohydrate metabolism	Small molecule biochemistry
Top 3 transcription regulators		
1	FOXO1	PPARA
2	NR5A2	PPARGC1A
3	EPAS1	NR4A1

*Abbreviations: EPAS1*, endothelial PAS domain-containing protein 1, *FOXO1* forkhead box protein O1, *NR4A1* nuclear receptor subfamily 4 group A member 1, *NR5A2* nuclear receptor subfamily 5 group A member 2, *PPARA*, peroxisome proliferator-activated receptor alpha, *PPARGC1A* PPAR gamma coactivator 1-alpha

### LF-specific, HF-specific, meal-responsive, HF-delay and LF-HF different genes

A significant canonical pathway, namely extracellular signal regulated kinase 5 (ERK5) signaling, and top three transcriptional regulators for the LF-specific genes were reflected as the top molecular and cellular functions (Table 2 and Additional file 2: Table S2).

**Table 2:** Significantly modulated canonical pathways, molecular and cellular functions, and transcription regulators for low fat (LF)-specific, high fat (HF)-specific, meal-responsive, HF-delay, and LF-HF-different genes

	LF-specific genes	HF-specific genes	Meal-responsive genes	HF-delay genes	LF-HF different genes
Top 3 canonical pathways					
1	ERK5 signaling	Calcium signaling	Aldosterone signaling in epithelial cells		Glycerolipid metabolism
2		LXR/RXR activation	Protein ubiquitination pathway		Actin cytoskeleton signaling
3		LPS/IL-1 mediated inhibition of RXR function			
Top 3 molecular and cellular functions					
1	Carbohydrate metabolism	Lipid metabolism	Cellular function and maintenance	Lipid metabolism	Lipid metabolism
2	Cellular function and maintenance	Small molecule biochemistry	Post-translational modification	Molecular transport	Small molecule biochemistry
3	Cellular growth and proliferation	Molecular transport	Protein folding	Small molecule biochemistry	Molecular transport
Top 3 transcription regulators					
1	CREB1	PPARA	FOXO1	RBPJL	NR5A2
2	CEBPB	SMAD3	AIP	PTF1A	RBPJL
3	FOXO1	PPARGC1A	HIF1A	MLX	Pdx1

*Abbreviations:* *AIP* aryl-hydrocarbon receptor-interacting protein, *CEBPB* CCAAT/enhancer-binding protein beta, *CREB1* cAMP responsive element binding protein 1, *ERK5* extracellular signal regulated kinase 5, *EPAS1* endothelial PAS domain-containing protein 1, *FOXO1* forkhead box protein O1, *HIF1A* hypoxia-inducible factor 1 alpha subunit, *IL-1* interleukin 1, *LPS* lipopolysaccharides, *LXR* liver X receptor, *MLX* Max-like protein X, *NR4A1* nuclear receptor subfamily 4 group A member 1, *NR5A2* nuclear receptor subfamily 5 group A member 2, *Pdx1* pancreatic and duodenal homeobox 1, *PPARA* peroxisome proliferator-activated receptor alpha, *PPARGC1A* PPAR gamma coactivator 1-alpha, *PTF1A* pancreas transcription factor 1 subunit alpha, *RBPJL* recombination signal binding protein for immunoglobulin kappa J region-like, *RXR* retinoid X receptor

The top three molecular and cellular functions for the HF-specific, HF-delay and LF-HF different genes were similar as the HF-responsive genes (Table 2). However, there was no common transcriptional regulator between the HF-specific genes (Additional file 2: Table S3) and HF-delay and LF-HF different genes (Additional file 2: Tables S4 and S5, respectively). Expression of the top transcriptional regulator for the HF-specific genes, PPARα, was significantly higher in the HF-3 h condition than in the LF-3 h condition (Fig. 2).

The meal-responsive genes were related to cellular function and maintenance, post-transcriptional modification, and protein folding (Table 2). The top transcriptional regulator was FOXO1 (Table 2 and Additional file 2: Table S6), and its expression was higher in fed condition (HF-3 h plus LF-3 h) compared to the fasting condition.

The functional distribution analysis of significantly modulated genes in each gene expression pattern indicates that both HF-delay and LF-HF different genes had a higher proportion of genes related to digestion (Fig. 1). In addition, LF-HF different genes had also a higher proportion of genes related to hormone/peptide (Fig. 1).

### SAGE and microarray

As shown on the Additional file 1: Figure S2, SAGE methods revealed a lower number of modulated transcripts in the receptor, signaling, and RNA/DNA processing but a higher number of transcripts of the cytoskeleton, and others/unknown. On the other hand, microarray detected a higher number of transcripts in both signaling and RNA/DNA processing, as well as a lower number of transcripts of the cytoskeleton and others/unknown functions.

The top three molecular and cellular functions detected by SAGE (lipid metabolism, molecular transport, and small molecule biochemistry) were also detected as the top four by microarray, and both SAGE and microarray revealed lipid metabolism and development related networks within the top three networks (Additional file 2: Table S7). In the SAGE-detected genes, 53% of identified transcription factors were represented in those of the microarray detected genes or 63% for vice versa (Additional file 2: Tables S8 and S9).

Significantly modulated genes from the SAGE analysis have been published [5]. The genes from the microarray analysis were presented in the Additional file 2: Table S10.

## Discussion

As expected, IPA revealed that “lipid metabolism” represents the main characteristic of both HF-responsive and HF-specific genes. Previous transcriptomic studies of the small intestine have used only microarray analysis and LF diet as a control [6–11], which will not allow a direct comparison with our data. However, most of the studies have pointed “lipid metabolism” as a key modulated function after several wks of HF feeding [7–11]. Our results and those of de Wit et al. [8–10] emphasize PPARα as a principal transcriptional regulator after a HF-meal ingestion. Indeed, the higher expression of PPARα protein after a HF meal was confirmed via a comparison with LF condition. Moreover, de Vogel-van den Bosch et al. [20] have also demonstrated, using PPARα-null mice, that PPARα is an important factor controlling expressions of intestinal barrier genes (multiple transmembrane transporter genes) following fatty acids (FA) ingestion. In the current results, molecular transport was placed at the top three of the molecular and cellular functions both in the HF-responsive and HF-specific genes. An average of 11% of the molecules found in the first and second networks contained these genes after the HF-meal ingestion, whereas no gene was found in the top two networks after the LF-meal ingestion (Additional file 1: Figures S3-S6). Furthermore, data from Steegenga et al. [11] have shown that lipid metabolism and small molecule biochemistry are the two major molecular and cellular functions after two wks of HF intake, whereas these two were in the top three in our acute HF-feeding results (both in the HF-responsive and HF-specific genes). Therefore, the previous results from the long-term HF feeding [7–11] and acute FA ingestion [20] support well our acute HF-feeding data.

Our study demonstrated that “carbohydrate metabolism” was one of the main characteristics after the LF meal ingestion, and FOXO1 and cAMP response element binding protein 1 (CREB1) as a principal transcriptional regulator for the LF-responsive and LF-specific genes, respectively. Both FOXO1 and CREB have been reported as two key transcriptional regulators for hepatic gluconeogenic program [21]. Under fasting condition, increased secretion of pancreatic glucagon triggers activation of protein kinase A (PKA), which phosphorylates CREB, leading to an increased expression of gluconeogenic genes such as glucose 6-phosphatase (G6Pase) [21]. Both in the liver and small intestine, Gautier-Stein et al. [22] have demonstrated that CREB binds to the G6Pase promoter after fasting but not in the postprandial state. On the other hand, FOXO1 stimulates G6Pase promoter activity through insulin response element (IRE) and increases its rate of transcription [23]. However, when phosphorylated, it is excluded from the nucleus [24], where it is then ubiquitinated and degraded [25]. After a high-carbohydrate-diet feeding, elevated blood glucose stimulates insulin secretion, which leads to the activation of insulin signaling pathways in the liver. The IRE mapped on the promoter of G6Pase is critical in mediating the insulin/Akt-dependent inhibition of gene expression in hepatic gluconeogenesis [23, 26]. In the present study, higher expressions of FOXO1 and phosphorylated CREB1 (a trend, *P* = 0.058) were seen in the fasting condition than in the LF-3 h condition, suggesting an elevated gluconeogenic pathway in the duodenum of fasting mice. In addition to the metabolism, FOXO transcription factors regulate cellular differentiation, growth, survival, cell cycle, stress and tumor suppression pathways [27]. Together with nuclear receptor subfamily 5 group A member 2 (NR5A2), which has emerged as a key regulator of intestinal function such as cell renewal and local immune function [28], these top 2 transcriptional regulators represent other molecular features of the LF-responsive genes, cellular function and maintenance as well as cell death. To our knowledge, there is no global investigation into the LF-responsive genes, since LF diet has been often used as a control [6–11]. Therefore, this is the first study reporting the characteristics of the LF-responsive genes with fasting condition as reference.

The present study identified ERK5 as a significant canonical pathway of the LF-specific genes. ERK5 signaling plays important roles in many cellular processes including cell proliferation, differentiation, survival and apoptosis by activating transcription factors including CREB1. CREB1 is known to interact with CCAAT/enhancer-binding protein beta (CEBPB, the second transcriptional regulator) which regulates genes involved in immune and inflammatory responses as well as maintenance of muscle function via macrophages [29, 30]. Together with the third transcriptional regulator FOXO1, these three transcriptional regulators and canonical pathway reflect the top three molecular and cellular functions, namely, carbohydrate metabolism, cellular function and maintenance, and cellular growth and proliferation.

Both HF-delay and LF-HF different transcriptomes were characterized by a higher proportion of genes related to digestion, and there were three common transcriptional regulators between the HF-delay and LF-HF different genes, namely recombination signal binding protein for immunoglobulin kappa J region-like (RBPJL), pancreas specific transcription factor 1a (PTF1A) and NR5A2. The RBPJL and PTF1A are transcription factors involved in the maximal production of digestive enzymes [28]. NR5A2 co-regulates an exocrine pancreas-specific transcriptional network of digestive function [22]. Thus, these common transcriptional regulators reflect well the common physiological function between the HF-delay and LF-HF different genes, “digestion”. Moreover, four (among seven) target molecules of these transcription factors in our data set, namely carboxyl ester lipase (*Cel*), chymotrypsinogen B2 (*Ctrb2*), trypsin 4 (*Try4*) and carboxypeptidase A2 (*Cpa2*), encode pancreatic digestive enzymes. The significance of these genes expressions remains unknown, however, the pancreatic digestive enzymes have been already reported to be expressed in normal epithelial cells of the duodenum [31].

In addition, the LF-HF different genes were also characterized with a higher proportion of genes related to hormone/peptide, such as islet amyloid polypeptide (*Iapp*), insulin I (*Ins1*) and insulin II (*Ins2*). After excluding the three transcriptional regulators common to LF-HF and HF-delay different genes, the remaining three transcriptional regulators in the top seven of this classification were related to pancreatic development and/or exocrine/endocrine pancreas-specific transcriptional network. More specifically, the pancreatic and duodenal homeobox 1 (PDX1) is a transcription factor necessary for pancreatic development [11, 32]. PDX1, v-maf musculoaponeurotic fibrosarcoma oncogene homolog A (MAFA) and homeobox protein Nkx-2.2 (NKX2-2) are necessary for β-cell maturation [32–34]. Furthermore, MAFA is a transcription factor for the insulin gene [35]. These transcriptional regulators represent the second characteristic of the physiological functional classification, “hormone/peptide”.

To our knowledge, there is no previous transcriptomic study investigating and analyzing the meal-responsive genes the way we did. Indeed, our data showed that the main molecular and cellular functions as well as the canonical pathway of meal-responsive genes are related to cellular function and maintenance as well as aldosterone signaling in epithelial, respectively. Aldosterone regulates electrolyte and water balance through its effects on ion transport in the epithelial cell. Ion channels and transporters play a critical role in ion and fluid homeostasis and thus in normal animal physiology. Our data indicate that only this classification had a higher number of significant transcriptional regulators which contain heat shock proteins (HSP) as target molecules (data not shown, Chi^2^ = 15.9). This reflects an importance of protein folding in the cells of duodenum after a meal ingestion, which is concordant with the third molecular and cellular function of meal-responsive genes, “protein folding”.

The gastrointestinal system plays a central role in immune system homeostasis [36]. It is the main route of contact with the external environment and is overloaded every day with exogenous stimuli, including dangerous pathogens and toxic substances [36]. Indeed, the small intestine is a xenobiotic-metabolizing organ [24]. Within this context, our transcriptional regulation analysis of the meal-responsive genes also highlighted the importance of xenobiotic-metabolism, namely aryl hydrocarbon receptor interacting protein (AIP) and aryl hydrocarbon receptor (AHR). In contrast to the meal-responsive genes, Van den Bosh et al. [25] have reported that fasting (12-24 h) modulates the expression of genes related to the xenobiotic-metabolism (phase I metabolic enzyme). In addition, they also reported changes in ATP binding cassette (ABC) superfamily of transporters (Abca1 and Abcg8) as a consequence of fasting [25]. When we compared the proportion of modulated genes related to transporters and phase I/II metabolic enzymes, the meal-responsive genes had a higher proportion of ABC transporters compared to other classifications (data not shown, Chi^2^ = 10.5). Although Van den Bosh et al. [25] have only focused on genes related to transporters and phase I/II metabolic enzymes, and used the AIN-93 M diet (LF diet) and fasted up to 24 h as well as whole small intestine instead of duodenum mucosa, their results supports well our data.

Wang et al. [37] have reported that dietary carbohydrate source (sucrose vs cornstarch, 52.3% dietary weight) affects gene expression profile of intestinal epithelium in mice, primarily metabolic pathways related to carbohydrate metabolism. In our study, sucrose content in the LF and HF meals are 33% and 9% weights, respectively, whereas glucose polymer contents are 35% and 18%, respectively. Moreover, LF groups consumed 2.7 to 4.1 times more sucrose than the corresponding HF groups, whereas only 1.4 to 2.3 times more glucose polymer was ingested in the LF groups [5]. On average, 1.6 times less sucrose than glucose polymer was consumed in HF-fed mice compared to the corresponding LF-fed mice. These may influence the results of present study. When we compared 95 individual genes listed in their study [37] to ours (896 transctipts), only two genes (namely growth factor receptor-bound protein 7 and stearoyl-Coenzyme A desaturase 2) from the microarray data and one gene (aldolase 1, A isoform) from the SAGE data were overlapped, representing 0.3% of our data set. Therefore, the effects should be minimum. In addition, since Wang et al. investigated long-term (10 wks) feeding effects and only used microarray data, the results cannot be compared directly. However, both studies agree with carbohydrate metabolism as one of the most modulated pathways after a high-carbohydrate diet.

SAGE and microarray use different approaches to detect, identify and quantify transcripts. SAGE method is based on the recognition of specific mRNA by the sequencing of short expressed tags (SAGE tags) that have been converted to cDNA, linked into concatemers and cloned [38]. The frequency of a SAGE tag in a biological sample is a precise measure of the amount of mRNA generated from a gene because all tags are simultaneously amplified without alteration in their relative proportions and without reference to an internal standard. However, cDNA without a specific restriction site such as *NlaIII* do not generate tags, and a large number of tags must be sequenced to observe significant differences between groups for low abundance mRNA [39]. In contrast to the SAGE, microarray technology is not limited by cDNA sequencing or by the presence of a restriction site in a transcript. Microarray is used to simultaneously detect a large number of mRNA species by their hybridization to nucleotide probes, which are synthetized in situ*,* on a chip [40]. Hybridization intensities can be used to compare mRNA between experimental groups, but microarray does not give an absolute estimate of genes expression level. In the present study, SAGE detected more transcripts in cytoskeleton whereas microarray detected more in signaling and RNA/DNA processing. Moreover, only 29 gene species (68 transcripts, 8%) in 896 modulated transcripts by the LF- and/or HF-meal ingestion were commonly modulated in both SAGE and microarray analysis, which demonstrates that SAGE and microarray detect different transcript species (92% of total modulated transcripts). However, only a negligible number of transcript (*n* = 1, 0.1%) reveals unexplainable contradicted result between them: Majority (20 genes) showed a similar or exactly the same expression pattern, whereas only two genes had opposite results and one of which detected by microarray does not contain SAGE tag, suggesting a different spliced product from the same gene. Even though SAGE and microarray detected different transcript species, majorities of the transcriptional regulators, the top five molecular and cellular functions, and the top three networks were represented in both methods. These results suggest that SAGE and microarray lead to the similar conclusion when the transcripts are globally analyzed. Therefore, SAGE and microarray complement each other, and combining the results will identify more variety of transcripts species that cannot be detected by SAGE or microarray alone.

## Conclusions

Our transcriptomic analysis using SAGE and microarray data highlighted lipid metabolism (PPARα) as the first molecular and cellular function (transcriptional regulator) of the HF-responsive and HF-specific genes. On the other hand, the first molecular and cellular functions of the LF-responsive and LF-specific genes as well as meal-responsive genes were carbohydrate metabolism (FOXO1 and CREB1, respectively) as well as cellular function / maintenance (FOXO1), respectively. We also characterized the common physiological function of the HF-delay and LF-HF different genes as digestion. Importantly, our results will contribute to the understanding of the LF and HF meal responsive mechanisms in the intestine and might allow the development of novel therapeutic approaches for obesity and the related disorders and disease. Indeed, PPARα deficient mice fed a HF diet exhibit an obese-phenotypic characterization [41] whereas an activation of PPARα transcriptional activity leads to protections from adiposity and insulin resistance [37, 42], which shows potential application for treatment of insulin resistance and obesity.

In addition, our pathway and network analysis indicate that the main gene expression in the duodenum shifts toward metabolisms corresponding to the major macronutrients ingested within 3 h after a meal and that there is a delay of digestion-related-genes modulation after a HF meal ingestion. These knowledges of the direct link between post-prandial duodenum gene expression and ingested meal as well as the delayed responses become useful for nutritionists and clinicians who would need to adjust the diet of their patients depending on their therapies. Since our study is about the short-term effects (up to 3 h), such aspect will be more important for drugs that are taken after meals and during the period when diet-induced metabolic variations are still active. For instance, a patient under therapies should avoid the diet that could deactivate the drug or prevent the metabolism of drug, especially if it is a prodrug, which could lead to either a loss of therapeutic efficiency or induce a pharmacotoxicity. Moreover, our results could also allow the nutritionists to better optimize the diet of patients suffering from divers metabolic diseases by considering the possible biochemical outcomes of the interactions between the ingested diet and the pathways induced by that diet depending on its ratio of lipids.

## Additional files

Additional file 1: Figure S1. Q_RT-PCR confirmation of SAGE and microarray. **Figure S2.** Comparison of the transcripts number detected in each functional classification between the serial analysis of gene expression (SAGE) and microarray. **Figure S3.** First network of the LF-responsive genes. **Figure S4.** Second network of the LF-responsive genes. **Figure S5.** First network of the HF-responsive genes. **Figure S6.** Second network of the HF-responsive genes. (DOCX 412 kb)

Additional file 2: Table S1. Antibody information for western blot. **Table S2.** LF-specific transcriptional regulators. **Table S3.** HF-specific transcriptional regulators. **Table S4.** HF-delay transcriptional regulators. **Table S5.** LF-HF different transcriptional regulators. **Table S6.** Meal-responsive transcriptional regulators. **Table S7.** Comparison between the serial analysis of gene expression (SAGE) and microarray in the molecular and cellular functions, networks, and transcription regulators after low-fat and high-fat meal ingestion. **Table S8.** Transcriptional regulators detected by SAGE. **Table S9.** Transcriptional regulators detected by microarray. **Table S10.** Normalized microarray data. (XLS 32 kb)

## Abbreviations

ABC: ATP binding cassette
AHR: Aryl hydrocarbon receptor
AIP: Aryl hydrocarbon receptor interacting protein
CEBPB: CCAAT/enhancer-binding protein beta
*Cel*: Carboxyl ester lipase
*Cpa2*: Carboxypeptidase A2
CREB: cAMP response element binding protein
*Ctrb2*: *Chymotrypsinogen* B2
DF: Density of each lane on the film
DM: Density of each lane on the membrane
ERK5: Extracellular signal regulated kinase 5
FA: Fatty acids
FOXO1: Forkhead box protein O1
G6Pase: Glucose 6-phosphatase
HF: High-fat
HSP: Heat shock proteins
*Iapp*: Islet amyloid polypeptide
*Ins1*: Insulin I
IPA: Ingenuity Pathways Analysis
IRE: Insulin response element
LF: Low-fat
MAFA: v-maf musculoaponeurotic fibrosarcoma oncogene homolog A
NKX2-2: Homeobox protein Nkx-2.2
NR5A2: Nuclear receptor subfamily 5 group A member 2
PC: Positive control
PDX1: Pancreatic and duodenal homeobox 1
PKA: Protein kinase A
PPAR: Peroxisome proliferator-activated receptor
PTF1A: Pancreas specific transcription factor 1a
Q_RT-PCR: Quantitative real-time PCR
RBPJL: Recombination signal binding protein for immunoglobulin kappa J region-like
SAGE: Serial analysis of gene expression
*Try4*: Trypsin 4
