# Supplementary material for: Identification of the principal transcriptional regulators for low-fat and high-fat meal responsive genes in small intestine
Source: Nutr Metab (Lond). 2017 Oct 23;14:66. doi: 10.1186/s12986-017-0221-3 (PMC5654052; doi:10.1186/s12986-017-0221-3)
Supplement: Supplementary file 1 — Q_RT-PCR confirmation of SAGE and microarray. Figure S2. Comparison of the transcripts number detected in each functional classification between the serial analysis of gene expression (SAGE) and microarray. Figure S3. First network of the LF-responsive genes. Figure S4. Second network of the LF-responsive genes. Figure S5. First network of the HF-responsive genes. Figure S6. Second network of the HF-responsive genes. (DOCX 412 kb) [file 12986_2017_221_MOESM1_ESM.docx]

**Supplemental Figure 1: Q_RT-PCR confirmation of SAGE and microarray.**

Each gene expression data from the SAGE, microarray and Q_RT-PCR was converted to the percentage of the fasting levels. *Significant difference from the fasting condition (*P*<0.05): The statistical analysis of SAGE and Q_RT-PCR data have been described in reference 5.

Abbreviations: Referred to the National Center for Biotechnology Information (NCBI) Entrez Gene (<http://www.ncbi.nlm.nih.gov/sites/entrez?db=gene>).


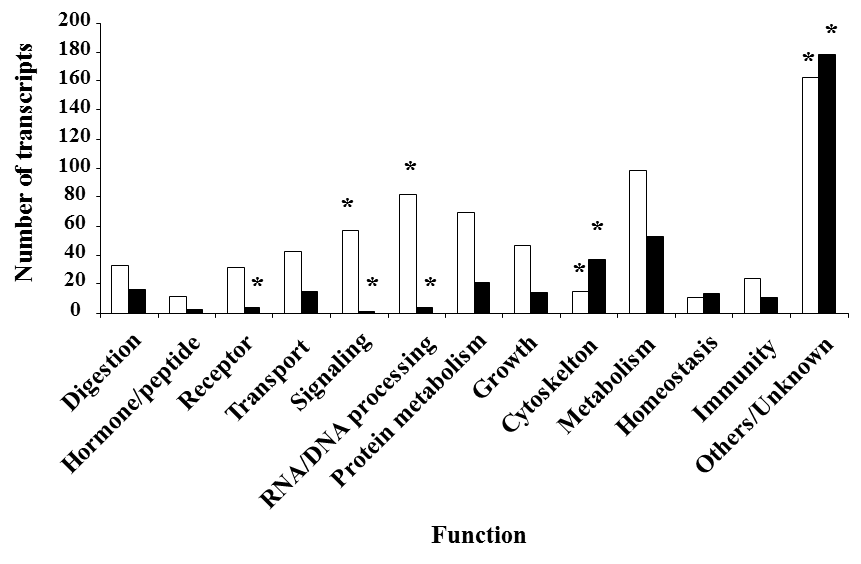


**Supplemental Figure 2: Comparison of the transcripts number detected in each functional classification between the serial analysis of gene expression (SAGE) and microarray.**

*Significant difference from the expected distribution of number of modulated transcripts (*P*<0.05); open bar, microarray; closed bar, SAGE.


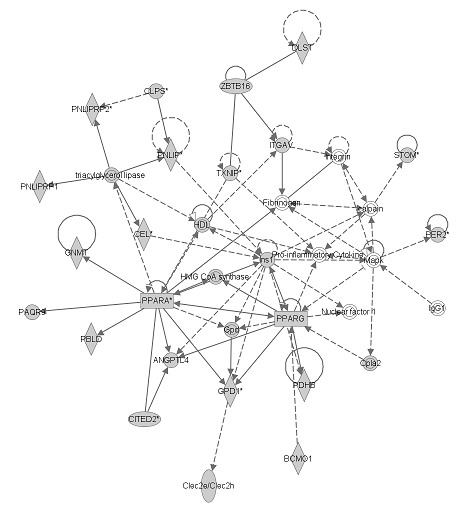


**Supplemental Figure 3: First network of the LF-responsive genes.**

Abbreviations: Refered to the National Center for Biotechnology Information (NCBI) Entrez Gene (http://www.ncbi.nlm.nih.gov/sites/entrez?db=gene).**
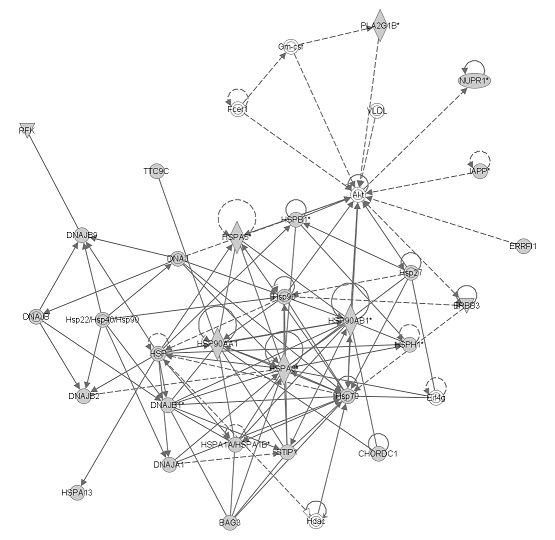
**

**Supplemental Figure 4: Second network of the LF-responsive genes.**

Abbreviations: Refered to the National Center for Biotechnology Information (NCBI) Entrez Gene (http://www.ncbi.nlm.nih.gov/sites/entrez?db=gene).**
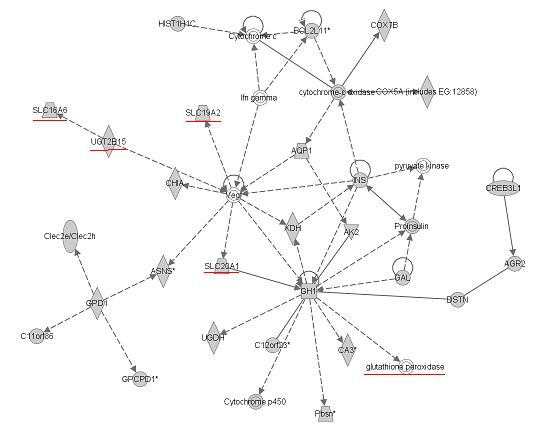
**

**Supplemental Figure 5: First network of the HF-responsive genes.**

Red underline: Intestinal barrier genes.

Abbreviations: Refered to the National Center for Biotechnology Information (NCBI) Entrez Gene (http://www.ncbi.nlm.nih.gov/sites/entrez?db=gene).


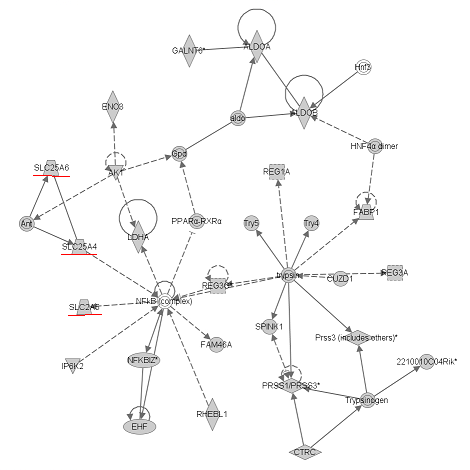


**Supplemental Figure 6: Second network of the HF-responsive genes.**

Red underline: Intestinal barrier genes.

Abbreviations: Refered to the National Center for Biotechnology Information (NCBI) Entrez Gene (http://www.ncbi.nlm.nih.gov/sites/entrez?db=gene).
